# Liposomal delivery of p-*ialB *and p-*omp25 *DNA vaccines improves immunogenicity but fails to provide full protection against *B. melitensis *challenge

**DOI:** 10.1186/1479-0556-8-5

**Published:** 2010-07-16

**Authors:** Nicola J Commander, James M Brewer, Brendan W Wren, Stephen A Spencer, Alastair P MacMillan, Judith A Stack

**Affiliations:** 1 Veterinary Laboratories Agency, Woodham Lane, New Haw, Addlestone, Surrey, KT15 3NB, UK; 2 Dr. James Brewer, Centre for Biophotonics, Strathclyde Institute of Pharmacy and Biomedical Sciences, University of Strathclyde, 27 Taylor Street, Glasgow G4 0NR, UK; 3 London School of Hygiene and Tropical Medicine, Keppel Street, London WC1E 7HT, UK

## Abstract

**Background:**

We have previously demonstrated protective efficacy against *B. melitensis *using formulations of naked DNA vaccines encoding genes *ialB *and *omp25*. The present study was undertaken to further understand the immune response generated by the protective vaccination regimens and to evaluate cationic liposome adsorption as a delivery method to improve vaccine utility.

**Methods:**

The protective efficacy and immunogenicity of vaccines delivered as four doses of naked DNA, a single dose of naked DNA or a single dose of DNA surface adsorbed to cationic liposomes were compared using the BALB/c murine infection model of *B. melitensis*. Antigen-specific T cells and antibody responses were compared between the various formulations.

**Results:**

The four dose vaccination strategy was confirmed to be protective against *B. melitensis *challenge. The immune response elicited by the various vaccines was found to be dependent upon both the antigen and the delivery strategy, with the IalB antigen favouring CD4+ T cell priming and Omp25 antigen favouring CD8+. Delivery of the p-*ialB *construct as a lipoplex improved antibody generation in comparison to the equivalent quantity of naked DNA. Delivery of p-*omp25 *as a lipoplex altered the profile of responsive T cells from CD8+ to CD4+ dominated. Under these conditions neither candidate delivered by single dose naked DNA or lipoplex vaccination methods was able to produce a robust protective effect.

**Conclusions:**

Delivery of the p-*omp25 *and p-*ialB *DNA vaccine candidates as a lipoplex was able to enhance antibody production and effect CD4+ T cell priming, but was insufficient to promote protection from a single dose of either vaccine. The enhancement of immunogenicity by lipoplex delivery is a promising step toward improving the practicality of these two candidate vaccines, and suggests that this lipoplex formulation may be of value in situations where improvements to CD4+ responses are required. However, in the case of *Brucella *vaccine development it is suggested that further modifications to the candidate vaccines and delivery strategies will be required in order to deliver sustained protection.

## Background

Brucellosis is a worldwide zoonosis of considerable social and economic importance. In livestock the principal clinical outcome of brucellosis is abortion. In humans the disease manifests as a debilitating flu-like illness which, if left untreated, can persist to become chronic with a variety of unpleasant sequelae. The disease is largely considered to be an occupational zoonosis as natural human infection is acquired through direct contact with the organism and most usually associated with contact with infected animals or animal products. In addition, brucellosis is one of the most frequently reported laboratory acquired bacterial infections and *Brucella *spp., are also considered potential biothreat agents (For review [1]). Whilst Great Britain and a large proportion of the developed world are designated as Officially *Brucella *Free (OBF), a considerable number of countries remain endemic for this debilitating zoonosis. Most notably, sheep and goat brucellosis caused by *Brucella melitensis *is an intractable problem in large areas of the Mediterranean basin and Near East, and is the cause of significant economic livestock industry losses and human morbidity.

*B. melitensis *infection in small ruminants can be controlled by vaccination with a live attenuated *Brucella *vaccine (Rev.1) [2,3]. Although this 'attenuated' vaccine is effective when used appropriately, it remains sufficiently virulent so as to cause abortion in pregnant animals and active brucellosis in man. Moreover, the generation of anti-*Brucella *antibodies following vaccination means that, using current serodiagnostic tests, there are difficulties in differentiating vaccinated and protected animals from those with true virulent infection. Non-living vaccines (mainly killed bacterin preparations) have been used intermittently in the past but have been discredited due to poor protective efficacy, generation of inappropriate immune responses, and poor standardizations [4]. More recently, vaccines of this type have been revisted and are showing some success [5]. Given the importance of *Brucella *zoonosis and the current difficulties with current vaccines, the development of an efficacious non-living defined vaccine is imperative towards improving control of this economically significant zoonosis. DNA vaccine technology has been successful in overcoming some of the limitations of killed cell and subunit protein preparations and a number of reports have shown protective DNA vaccination against brucellosis in the murine model with relatively simple constructs encoding a single protective antigen [6-11]. Indeed we previously reported protective activity from two candidate DNA vaccines based upon *B. melitensis omp25 *and *ialB *genes [12]. However, naked DNA vaccination is known to be a relatively inefficient process and protection is rarely achieved following a single inoculation. Several strategies have been used to enhance the immunogenicity of various *Brucella *DNA vaccines. For example plasmid vectors have been used to deliver cytokines in addition to the protective antigen [13-15], and prime boost approaches have been reported with some success. For example Cassataro *et al *[16], reported moderate improvements to protective efficacy of a DNA vaccine through use of a heterologous prime boost strategy to deliver the BLSOmp31 chimeric DNA vaccine. However, thus far, none of the DNA vaccines described above have been shown to elicit significant levels of protection in the mouse model or target species after only a single immunisation. Munoz-Montesino *et al *[17] achieved modest protective efficacy from a single intrasplenic inoculation of mice with a DNA vaccine based upon Cu/Zn SOD, although equivalent quantities of this vaccine were not efficacious when delivered by the more usual, intramuscular, route. Interestingly, Saez *et al *[18] have recently demonstrated immunogenicity of the *Brucella *Cu/Zn SOD based DNA vaccine in cattle, indicating the potential of the DNA vaccine approach for *Brucella *to be carried through to the target species. Their vaccine was able to induce antigen specific T cell proliferation and an IgG1 isotype dominated antibody response, but protective efficacy was not investigated in these studies. Notably, the measured quantities of IFNγ, the essential mediator of protective immunity was relatively low in these studies, suggesting that modifications may be beneficial for more efficient or efficacious vaccination of livestock.

Different routes of delivery have been investigated to improve DNA uptake and *in vivo *expression including techniques such as *in-vivo *electroporation [19,20] and the use of microparticulate delivery systems [21], which have also been used with success specifically for brucellosis vaccination with acellular antigen extracts [5]. Encapsulation or surface adsorption of DNA to cationic liposome preparations has also been shown to be a simple method for improving the immune response to DNA vaccines [22,23]. Our own studies previously demonstrated a protective effect from DNA vaccines encoding the *B. melitensis *16 M genes *ialB *and *omp25 *when delivered as four discreet intramuscular inoculations given at three week intervals. Moreover, preliminary assessment of the vaccine induced immune responses suggested specific responses were elicited after fewer than four inoculations. Therefore, in this follow up study we aimed to assess the performance of a single dose of the candidate DNA vaccines, when delivered as either naked DNA or surface adsorbed to cationic liposomes (lipoplex). Protective efficacy and vaccine specific immune responses were measured in order to determine if this simple method of delivery via lipoplex could improve the efficiency of DNA vaccination, and thereby ultimately facilitate transfer of this research to the target animal.

## Materials and methods

### Experimental design and vaccine production

Plasmids encoding the *Brucella *proteins Omp25 and IalB were produced as described previously [11]. The immunogenicity and protective efficacy of the candidates was evaluated after either, (a) the known protective regimen of four doses of naked DNA vaccine, (b) a single dose of naked DNA vaccine or (c) a single dose of lipoplexed DNA vaccine.

#### Plasmid based vaccines

DNA vaccines p-*omp25 *and p-*ialB *were produced as described previously [11]. Briefly, the *ialB *and *omp25 *genes were amplified by PCR and modified to encode a 5' Kozac signal sequence to facilitate eukaryotic expression. PCR products were cloned into the pCR3.1 vector (Invitrogen) and the pTargeT (Promega) expression vector (*omp25 *product only). Sequence fidelity and orientation was checked through sequencing and restriction enzyme fragmentation and *in vitro *expression from the plasmids was verified following transfection of Cos7 cells as previously described. Bulk stocks of endotoxin free DNA vaccine plasmids and vector control plasmid (pcDNA3.1) in 0.1 M PBS were produced for *in-vivo *studies by Plasmid Factory GmbH, (Bielefeld, Germany). For this study plasmids were generated using the pCR3.1 (Invitrogen) or pTargeT (Promega) backbones. Preliminary investigations suggested that the kinetics and quantity of *in-vitro *expression for the Omp25 protein was uninfluenced by the plasmid backbone (data not shown). Thus, for the investigations described herein, a preparation consisting of a 1:1 mixture of both plasmid types was used as the p-*omp25 *vaccine. p-*ialB *preparations were based upon the pCR3.1 backbone only.

#### Lipoplex production

Lipid vesicles were prepared from 1-monopalmitoyl glycerol, cholersterol, stearyl amine and cetyl trimethyl ammonium bromide (CTAB (All from Sigma UK, Poole, Dorset)) in the molar ratio 5:4:1:1, by the methods described previously [24]. The p-*ialB*, p-*omp25 *and pcDNA3.1 plasmids were surface adsorbed to the cationic vesicles immediately prior to vaccination. Briefly, 2 ml of plasmid DNA at 1 mg/ml was added dropwise to an equal volume of liposome preparation. The plasmid:liposome mixture was gently mixed using a Denley Rotary Cell Mixer for 30 minutes at 25°C, and then centrifuged at 2000 rcf to sediment the complexes. 2 ml of supernatant (SN) was removed and set aside for retrospective analysis of DNA content by spectrophotometry (A_260 _determination) and agarose gel electrophoresis. This data was used to determine whether adsorption of DNA to liposomes had been successful: absence of detectable DNA in the SN indicating that DNA was complexed to the liposomes. Successfully adsorbed liposome DNA complexes were resuspended in the remaining SN by gently mixing, and used for vaccination within one hour of production. Control solutions of non DNA complexed liposomes were treated identically with 0.1 M PBS.

#### Vaccination and challenge experiments

Groups of mice (4 < n < 10) were intramuscularly inoculated with either naked DNA (p-*ialB*, p-*omp25 *or plasmid control pcDNA3.1) at 100 μg/mouse/dose, or an equivalent quantity of the DNA surface adsorbed to cationic liposomes (L-p-*ialB*, L-p-*omp25 *and L-pcDNA3.1 respectively). Uncomplexed liposomes (without adsorbed DNA) and PBS were administered in equivalent dosing volumes to control groups. In each study protective efficacy of the candidate vaccines was compared to that of the live attenuated strain *B. melitensis *Rev.1. Rev. 1 was administered subcutaneously as a single dose of approximately 2 × 10^5 ^CFU per mouse to the control groups. For assessment of protective efficacy mice were challenged with approximately 1 × 10^4 ^CFU *B. melitensis *strain 16 M given via intraperitoneal inoculation at 30 days post-vaccination. *B. melitensis *16 M and Rev.1 strains were obtained from the VLA culture collection and propagated and prepared for use as described previously [11]. The number of bacteria present in the spleens of the mice at 15 ± 1 days post challenge was used to compare the protective effects of the candidates and controls. Splenic homogenates were serially diluted and cultured on TSA (+5. I.U Penicillin) media at 38°C, with 10% CO_2 _for 5 - 7 days. Bacterial load per group was compared using one-way ANOVA of log transformed data.

### Immunological response assessments

#### ELISA for measurement of serological responses to vaccination

Antibody responses were measured in colorimetric ELISA against *B. melitensis *16 M whole cell antigen using protocols and reagents described previously [11]. The sera was also assessed against recombinant Omp25-GST, GST and IalB proteins, in an identical ELISA format. Purification of recombinant IalB and Omp25 was performed by Lionex GmbH (Germany), and these antigens were coated on Nunc Polysorb plates at concentrations of 10 μg/ml, 10 μg/ml and 15 μg/ml respectively. The response of individual mice at each time point was assessed. For the measurement of Omp25 specific responses the OD of the GST reaction was subtracted from that of the corresponding Omp25-GST reaction, to eliminate responses specific to the GST tag on the recombinant protein. Positive responses were recorded from sera reading a greater OD than the assay Cut Off (CO). CO was determined as Mean + 3 X standard deviation (SD) of plate specific negative control samples (Normal mouse sera at 1/40 dilution) in ELISA.

Following assessment of individual mouse responses, pooled samples were created for each group. The specific IgG1 and IgG2a titres were obtained from the pooled serum samples from each group. Endpoint titres for each group at each time point were determined as the dilution at which the sample OD first becomes lower than the plate Cut-off (CO) value.

#### Measurement of antigen specific IFNγ production

IFNγ production was measured by ELISPOT following specific antigen stimulation of splenocytes from vaccinated mice. Specific stimulatory antigens included Brucellergene™ (Synbiotics Europe, Merial, France), a commercially available preparation of cytosolic antigens derived from rough strain *B. melitensis *B115. Brucellergene™ was dialysed against PBS prior to use to remove the preservatives, and prepared to a 40 μg/ml final concentration in stimulation assays. Specific recombinant IalB [used at 15 μg/ml final concentration], recombinant GST [10 μg/ml] or recombinant Omp25-GST [10 μg/ml] were also used as stimulating antigens depending upon the particular investigation. Concanavalin A at 5 μg/ml final concentration was used as a mitogen control in these assays, and antigen free media (DMEM complete) was used as a no stimulation control.

Assays were conducted at three weeks post-vaccination and two weeks post-challenge in order to compare the antigen specific immune response of candidates and controls.

For each ELISPOT investigation (conducted three weeks after completion of the selected vaccination protocol), splenocyte preparations from five animals per group were pooled and processed to produce CD4+ depleted cell populations and CD8+ depleted cell populations. Anti-mouse CD4+ (L3T4) and anti-mouse CD8+ (Ly-2) magnetic beads (Miltenyi Biotech) were used to bind the CD4+ and CD8+ expressing cells in the total splenocyte population and depletions were carried out using Midi-Macs (Miltenyi Biotech) cell separation technology. Briefly, the total splenocyte concentration was adjusted to 1 × 10^9 ^cells per ml in ice cold FacsFlow (FF) buffer (BD Biosciences), and three separate replicates of the cell sample were created. One replicate was treated with anti-CD4+ beads and another with anti-CD8+ beads (100 μl of beads was used per ml of cell suspension). The third replicate served as a 'no bead' control. Samples were incubated on ice for 30 minutes with occasional gentle mixing. Midi-Macs™ LS columns (Miltenyi-Biotech) were equilibrated with ice cold FF and positioned in the magnetic clamps. The cell preparations were applied to the LS columns and the fall through fraction collected in clean sterile tubes over ice. The columns were washed through with three volumes of ice cold FF and the total eluate collected and washed by centrifugation in ice cold FF. The final preparations, "CD4+ depleted", "CD8+ depleted" or "Total/undepleted" were resuspended in a minimal volume of ice cold FF for enumeration and then supplemented with DMEM complete to a final concentration of 5 × 10^6 ^cells per ml for use in ELISPOT.

ELISPOT nitrocellulose membrane plates (Millipore) were prepared by coating with anti-IFNγ mAb (AN18) (MabTech, Sweden) at 15 μg/ml, overnight incubation at 4°C in coating buffer pH 9.6. Plates were washed twice in PBS and blocked for one hour with DMEM complete media at 37°C prior to incubation with cells and antigens at the concentrations described previously. Stimulated cultures were incubated (37°C, 5% CO_2_) for 24 ± 2 hours, loosely wrapped in aluminium foil. Following incubation cells were aspirated from the filter plates and the membrane washed four times with PBS-T wash buffer. Anti-mouse IFNγ biotinylated antibody (MabTech, Sweden) at 1 μg/ml in PBS-B (PBS + 1%BSA) was added and incubated at 25°C. Plates were washed four times with PBS-T prior to the addition of Streptavidin-alkaline phosphatase reagent (GE Biosciences) (1 in 1000 in PBS-B) and incubation at 25°C for one hour. Plates were then washed four times with PBS-T and then twice with distilled water, before application of 0.2 μM filtered BCIP/NBT (BCIP/NBT Fast Tabs, Sigma UK) solution for developing the spots. The reaction was halted by rinsing in distilled water as soon as the colour development in the ConA control stimulation wells was complete - with wells showing a confluent block of colour. Plates were then fumigated to ensure sterility and air dried before reading using an AID ELISPOT reader.

All animal work was approved by the VLA Ethics Committee, and in line with A(SP)A 1986 regulations.

## Results

### Immunological responses to vaccination and challenge

#### Serological response to vaccination

The serological response of the mice to vaccination was investigated by ELISA. Antigen specific IgG1 and IgG2a were measured from sera collected at three weeks post-vaccination. These data are presented in table 1.

**Table 1:** Humoral immune responses to vaccination

**Titre (and percentage of animals responding) of specific IgG1 and IgG2a antibodies in ELISA**.						
**ELISA**	**Omp25-GST**		**IalB**		**16M**	
	**IgG1**	**IgG2a**	**IgG1**	**IgG2a**	**IgG1**	**IgG2a**

p-*omp25 *[X1]	Neg	1/320 (60%)	ND	ND	Neg	Neg
p-*omp25 *[X4]	1/640 (100%)	1/1280 (100%)	ND	ND	1/420 (100%)	1/520 (100%)
L-p-*omp*25	1/320 (70%)	1/1000 (100%)	ND	ND	1/640 (70%)	1/1280 (100%)

p-*ialB *[X1]	ND	ND	Neg	Neg	Neg	Neg
p-*ialB *[X4]	ND	ND	1/5210 (100%)	1/5210 (100%)	1/5120 (100%)	1/5120 (100%)
L-p-*ialB*	ND	ND	1/270 (80%)	1/40*(10%)	1/500 (80%)	1/120 (10%)

pcDNA3.1 [X1]	Neg	Neg	Neg	Neg	Neg	Neg
pcDNA3.1 [X4]	Neg	Neg	Neg	Neg	Neg	Neg
L-pcDNA3.1	Neg	Neg	Neg	Neg	Neg	Neg

Uncomplexed Liposome	Neg	Neg	Neg	Neg	Neg	Neg

Rev.1a	Neg	Neg	Neg	Neg	Neg	Neg
Rev.1b	1/640 (100%)	1/640 (100%)	Neg	Neg	1/2560 (100%)	1/2560 (100%)

PBS	Neg	Neg	Neg	Neg	Neg	Neg

Rev.1a, serum sample taken at two weeks post-vaccination. Rev.1b serum sample taken at 12 weeks post-vaccination. Neg: All individual OD values below assay C/O. ND: Sample not tested. The number of individual sera per group for each assay n ≥ 10.

Data from all naked DNA vaccinated groups indicated development of a Th1 biased serological response. The four dose p-*omp25 *vaccine regimen resulted in detectable Omp25 specific IgG1 and IgG2a from 100% of mice, with titres of 1/640 and 1/1250 respectively. The single dose group generated a more modest antibody response with 60% of the mice noted to have Omp25 specific IgG2a antibodies. The titre of the sera pooled from this group was 1/320. Omp25 specific IgG1 antibodies were not detected from any mice in this group. A single dose of the lipoplex p-*omp25 *resulted in an overall stronger humoral immune response than the single dose naked DNA with 100% and 70% of vaccinates shown to have Omp25 specific IgG2a and IgG1 respectively. The titres for the pooled sera from this vaccine group were 1/320 and 1/1000, for IgG1 and IgG2a respectively. Thus, the delivery of the vaccine as a lipoplex appeared to enhance antibody production, and promote a more balanced IgG1/IgG2a antibody profile than naked DNA.

For the *ialB *based vaccines, the four dose regimen resulted in strong antibody responses with 100% of mice producing specific IgG1 and IgG2a. Group pooled sera titres were > 1/5000 for both isotypes. Unfortunately no antibody response was apparent after a single inoculation with naked DNA. However, a single dose lipoplex vaccination with this construct produced measurable IalB specific responses in 80% of mice (IgG1 response), albeit of a relatively low titre (1/270). One of the animals in the group (10%) was demonstrated to be positive in the IgG2a specific tests (OD > 0.25). However, in the pooled group titration this response was diluted such that the overall group response would be considered negative at 1/40.

Overall the data suggests that delivery of a single dose of DNA as a lipoplex resulted in an improvement in antibody production for both candidates, in comparison to that elicited by naked DNA. For example the lipoplex p-*omp25 *vaccine was able to induce an equivalent antibody response to that observed from the four dose regimen. More strikingly, a single inoculation of lipoplexed p-*ialB *elicited detectable antibody from mice whereas single dose naked DNA did not. However, the single dose lipoplex p-*ialB *vaccination was unable to elicit an equivalent antibody response to that achieved following four doses of naked DNA.

#### Cellular immune responses to vaccination and infection

Antigen specific IFNγ production was measured following *in vitro *stimulation with *Brucella *specific antigens (Brucellergene™, recombinant IalB, recombinant Omp25), and further information on the cellular origins of the IFNγ responses were derived from specifically isolated CD4+ and CD8+ depleted splenocyte populations.

**Figure 1:**
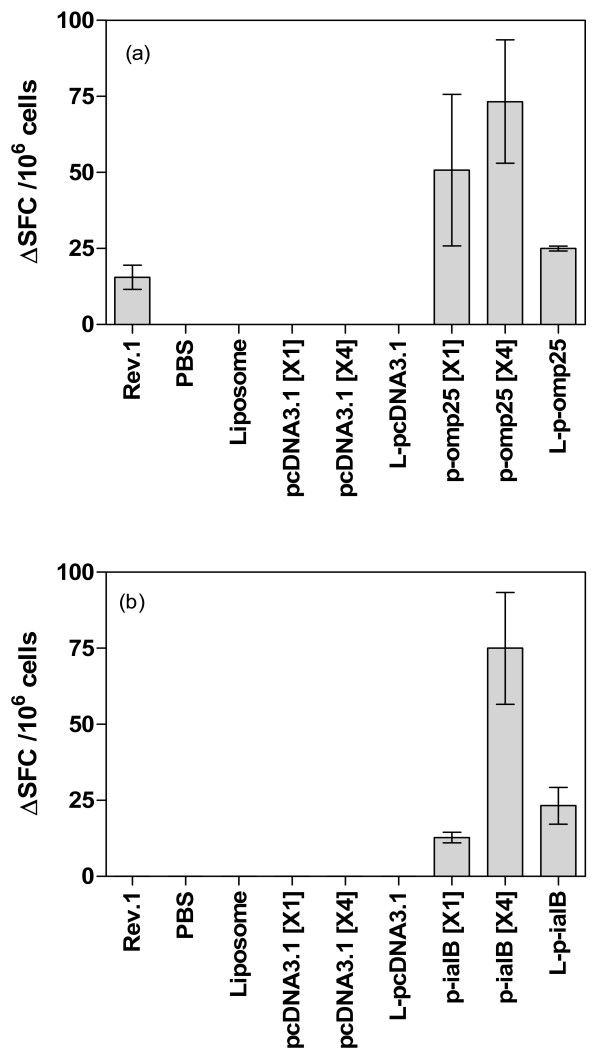
**Total ΔSFC per million cells detected in response to stimulation with (a) Omp25 antigen or (b) IalB antigen, for each of the vaccine groups. Measured from splenocytes harvested at three weeks post-vaccination**. (a) stimulation of splenocytes with Omp25 antigen (10 ug/ml). (b) Stimulation of splenocytes with IalB antigen (15 ug/ml). Bars represent the total ΔSFC detected per million splenocytes for each vaccine (Number of spots detected in stimulated sample - number detected in corresponding unstimulated sample). Error bars represent the standard deviation of replicate samples.

Figure 1 summarises the antigen specific IFNγ responses (total ΔSFC/10^6 ^cells) elicited by each of the different vaccination strategies. Table 2 summarises the data obtained from ELISPOT assays to measure IFNγ production from CD4+ or CD8+ depleted splenocyte populations from vaccinated mice. The data reveals that each vaccine regimen is capable of inducing antigen specific IFNγ production from *in vitro *restimulated splenocytes. However, there are notable differences, both quantitative and qualitative, between the responses elicited by the different regimens and vaccines.

**Table 2:** IFNγ ELISPOT data for the ialB and omp25 based vaccines showing the contribution of CD4+ and CD8+ T cells to the total response.

Vaccine	Net responsive cells		
	**Total cell population**	**CD4+ depleted cell population**	**CD8+ depleted cell population**

**Stimulation with Omp25 (10 μg/ml)**			

p-*omp25 *[X1]	50.8 ± 24.9	27.8 ± 2.3 [↓45%]	11.3 ± 2.3 [↓78%]
p-*omp25 *[X4]	73.3 ± 20.3	41.0 ± 5.2 [↓44%]	10.8 ± 3.5 [↓85%]
L-p-*omp25*	25.0 ± 0.77	2.8 ± 0.08 [↓89%]	13.0 ± 0.9 [↓48%]

**Stimulation with IalB [15 μg/ml]**			

p-*ialB *[X1]	12.75 ± 1.75	0	0
p-*ialB *[X4]	75.02 ± 18.37	11.0 ± 4.85 [↓85%]	36.25 ± 2.92 [↓52%]
L-p-*ialB*	23.25 ± 6.06	0	0

A comparison of Rev.1 based immunity and that generated by the two candidate vaccines is not entirely appropriate due to the differences in live versus subunit vaccination approaches, but can be used to benchmark the type of response required for effective protective efficacy. Rev.1 immunised animals produce *Brucella *specific IgG1 and IgG2a and a CD4+ T cell dominated IFNγ response. Responses from the Rev.1 vaccinated animals revealed antibody and IFNγ production in response to specific antigens Brucellergene™ and Omp25 but not to IalB, suggesting that this antigen does not have a major role to play in immunity generated by this vaccine.

Significant differences in IFNγ production were observed between the different vaccination strategies in the mice receiving the *omp25 *based DNA vaccines. The four dose naked DNA regimen (p-*omp25 *[X4]) was found to result in the strongest IFNγ response to Omp25 antigen The single dose of naked DNA also resulted in measurable IFNγ production which was not considered significantly different to that achieved by the four dose regimen. Notably, a single dose of lipoplex *omp25 *resulted in a lower total IFNγ response than the naked DNA approaches. Statistical analysis (Mann-Whitney test) did not reveal a significant difference in the quantity of IFNγ producing cells elicited between the single dose naked DNA and single dose lipoplex formulations (p > 0.05) or between the single dose naked DNA and multi-dose naked DNA regimen (p > 0.05), but did suggest a significant difference between the total IFNγ cells for multi-dose and single lipoplexed DNA regimens (p < 0.05). Overall, these data show that each *omp25 *based vaccine elicits detectable levels of IFNγ secreting cells and suggests that delivery of as a lipoplex does not notably augment the cellular response compared to that achieved by naked DNA vaccination.

CD4+ and CD8+ subset analysis for the *omp25 *based vaccines suggested that the CD8+ subset of the splenocyte population were responsible for the majority of observed IFNγ production in both the single or multi-dose naked DNA vaccinated groups. Whilst depletion of CD8+ cells from total splenocytes did not abrogate the detectable response there was a considerable decrease in the detectable SFC (85%), indicating the majority of IFNγ producing cells to be a CD8+ phenotype. However, when this vaccine was delivered as a lipoplex the cellular contributions appear to be altered with the depletion of CD4+ cells having the most dramatic effect upon detectable SFC, suggesting that whilst cellular responses are not quantitatively improved by lipoplex delivery there may be an effect on the priming of different subsets.

The multiple dose p-*ialB *naked DNA regimen resulted in a similar total of detectable antigen specific SFC to that observed for the equivalent delivery strategy with p-*omp25*. Relatively few antigen specific SFC were observed when a single dose p-*ialB *naked DNA vaccine was used, but lipoplex delivery of this vaccine prompted a modest increase in the number of SFC. The difference between single and multi-dose vaccine elicited SFC was considered significant (p < 0.05, Mann-Whitney test), suggesting multiple vaccinations are required for effective T cell priming. The difference in T cell response between single dose naked DNA and lipoplexed p-*ialB *was not found to be significant in these analyses. Nevertheless, the modest increase in SFC suggests that liposomal delivery of p-*ialB *has the potential to enhance the capacity of this vaccine for inducing an antigen specific cellular immune response.

For the four dose naked p-*ialB *protocol the depletion of CD4+ cells resulted in the most notable reduction of SFC (85%), suggesting that these cells were the principal producers of the IFNγ. This contrasts with the result from the p-*omp25 *vaccines where the CD8+ subset are dominant for IFNγ production. Unfortunately, in the single dose studies the depletion of either subset (CD4+ or CD8+) abrogated the detectable IFNγ response, and hence it is not possible to deduce whether one subset has a more prominent role to play in IFNγ production.

ELISPOT analysis of total splenocytes was also performed post-challenge, to determine whether the different vaccines or vaccination strategies resulted in different immune profiles during progression or clearance of infection. The data revealed the presence of IFNγ secreting cells following stimulation with Omp25 in all groups of animals. Relatively high numbers of SFC (> 25 SFC) were determined in all groups and in many cases automated plate reading revealed saturated responses. Saturation was estimated to be equivalent to ≥ 200 SFC. A relationship between the number of Omp25 specific SFC detected post-challenge and previous exposure to this antigen (through vaccination with the *omp25 *based preparations or Rev.1) was not demonstrable. In contrast, the post-challenge IalB stimulation data showed that only groups of animals that had been deliberately exposed to this antigen had significant IalB specific responses post-challenge. Significant production of IFNγ was apparent from the mice vaccinated with multi-dose (> 200 SFC) or single dose naked DNA (6.0 ± 0.62 SFC) or single dose lipoplex DNA (43.5 ± 3.2 SFC) but not from any of the non-immune control groups. These findings suggest that *ialB *vaccines have primed the immune response to this antigen which may be weakly expressed during the early stages of infection, but may be an important protective antigen.

### Demonstration of protective efficacy of the different vaccine regimens

Protective efficacy was measured in three experiments. Each individual study contained appropriate positive (Rev.1 immunised) and negative (PBS inoculated) control groups. The mice received 2.05 × 10^5 ^CFU per mouse (study 1), 2.26 × 10^5 ^CFU per mouse (study 2) and 1.95 × 10^5 ^CFU per mouse (Study 3) of Rev.1 vaccine control. At challenge mice received 2.16 × 10^4 ^CFU per mouse (study 1), 2.66 × 10^4 ^CFU per mouse (study 2), and 2.26 × 10^4 ^CFU per mouse (study 3), of *B. melitensis *16 M. Upon completion of all three experiments each individual study and the combined data was analysed to enable comparison of each of the various vaccine candidates and protocols. Direct comparison of the recovered bacterial load from the Rev.1 and (PBS) naïve controls of each experiment revealed no significant difference between studies (Two-way ANOVA and Dunnets post-test, p > 0.05) suggesting that the studies were consistent enough to permit qualitative inter-study comparison of efficacy of test candidates.

Table 3 shows the mean Log_10 _CFU per spleen *B. melitensis *16 M recovered from spleens of mice at 15 ± 1 day post-challenge. In all three studies the Rev.1 vaccine provided expected levels of protective effect, ranging between 2.04 and 3.75 PU across the studies. Similarly, the four dose vaccination regime for p-*ialB *or p-*omp25 *resulted in the expected statistically significant reduction in bacterial load in comparison with the naïve mice. The PU for the four dose naked DNA vaccination regimens ranged from 2.15 to 3.45 in these studies, indicating equivalent protective efficacy to Rev.1 in this model and confirming the findings of previous studies.

**Table 3:** The protective effect of vaccination with naked DNA or liposome formulated DNA.

Vaccine group	*Brucella *CFU per spleen	*Brucella *per spleen as a % of challenge dose	Protection units
**Experiment 1: A comparison of single dose naked DNA vaccine efficacy**			
Rev.1	2.95 ± 0.35*	67.8	2.04*
PBS	5.00 ± 0.05	114.8	0
pcDNA3.1 [X1]	4.56 ± 0.31	104.8	0.43
p-*omp25 *[X1]	4.45 ± 0.44	102.3	0.55
p-*ialB *[X1]	4.19 ± 0.42	96.4	0.80

**Experiment 2: p-*omp25 *[X4] compared with L-p-*omp25 *[X1]**			
Rev.1	1.47 ± 0.82*	33.2	3.35*
PBS	4.83 ± 0.29	109.2	0
pcDNA3.1 [X4]	4.80 ± 0.21	108.5	0.02
p-*omp25 *[X4]	1.40 ± 0.69*	31.7	3.42*
L-p-omp25 [X1]	4.01 ± 0.14*	90.8	0.81*
L-pcDNA3.1	4.86 ± 0.44	109.8	-0.03

**Experiment 3: p-*ialB *[X4] compared with L-p-*ialB***			
Rev.1	1.94 ± 0.77*	44.8	2.12*
PBS [X4]	4.10 ± 0.68	93.6	0
PcDNA3.1 [X4]	3.75 ± 0.36	86.1	0.32
p-*ialB *[X4]	1.91 ± 0.93*	44.0	2.15*
L-*p-ialB *[X1]	3.07 ± 1.08	70.5	1.00
L-pcDNA3.1	3.37 ± 0.58	77.5	0.70

Brucella CFU per spleen: Log Brucella CFU per spleen ± standard deviation.

Protection units = Log Brucella CFU per spleen of unvaccinated mice - Log Brucella CFU per spleen of vaccinated mice. % Challenge dose: (Log CFU Brucella per spleen/Log CFU challenge dose) × 100.

* indicates statistically significant reduction of Brucella CFU per spleen compared to PBS controls in the same study (One Way ANOVA analysis with Dunnets post-hoc test, p < 0.05)

A single dose of either the p-*ialB *or p-*omp25 *naked DNA vaccines resulted in a very slight reduction in bacterial load compared with the concurrent naïve control groups (0.80 and 0.55 PU respectively) but analysis did not reveal this reduction to be statistically significant. Furthermore, a similar value of 0.43 PU was obtained for the empty vector control in these studies, thereby indicating that a single dose of either p-*ialB *or p-*omp25 *delivered as naked DNA was unable to provide a significant antigen specific protective effect.

The single dose lipoplexed vaccines resulted in a slight reduction in bacterial load in comparison to the naïve controls. In these studies the difference between vaccinated animals and naïve (PBS) animals amounted to 1.0 PU for L-p-*ialB *and 0.81 for L-p-*omp25*. In both cases the PU measured for the lipoplex version of the single dose vaccine exceeded that of the equivalent dose of naked DNA, suggesting a possible improvement in vaccine performance through delivery as a lipoplex. However, these differences were not determined to be statistically significant (ANOVA with Dunnets post-hoc test, p > 0.05). Furthermore, the comparison of bacterial loads for the naïve controls and the lipoplex samples did not reveal a statistically significant protective effect from lipoplexed vaccines.

Overall, the protective efficacy investigations confirmed the findings that p-*ialB *and p-*omp25 *vaccines provide significant protective efficacy when delivered in a regimen of four discrete 100 μg inoculations given at 3 week intervals. Unfortunately, a single dose of these vaccines was unable to provide a robust or significant protective effect when delivered either as naked DNA or lipoplex. Notably, lipoplex delivery does appear to increase the protective efficacy of a single dose each vaccine but not to a statistically significant level.

## Discussion

Previous studies [11] indicated a protective effect from two candidate DNA vaccines based upon the *omp25 *and *ialB *genes of *B. melitensis *in the murine model of brucellosis. Protective efficacy was achieved after four separate 100 μg inoculations. This finding was confirmed in the present study. However, in order for these vaccines to be practical for use in livestock the number of inoculations and quantity of DNA required to elicit a protective response ideally needs to be reduced. The relatively poor immunogenicity of naked DNA vaccines is well established and considerable effort has been invested in assessment of vaccination protocols, formulations and strategies to improve their potency (for review see [25,21]. Lipoplex delivery of DNA is one such strategy which probably works through a combination of a potent adjuvant effect [26,27] and the presence of the lipid providing the plasmids with some protection against degradation by nucleases *in vivo*. Lipoplexing as a delivery strategy therefore has potential to improve antigen delivery to antigen presenting cells (APCs). To this end we chose to re-evaluate our two candidate DNA vaccines as single doses of naked DNA (100 μg per mouse) and an equivalent quantity of the DNA surface adsorbed to a novel formulation of cationic liposomes (lipoplex).

We found that each of our single dose vaccines was able to induce significant and appropriate antigen specific immune responses, albeit to a lesser extent than the multiple dose naked DNA strategy. Furthermore, the use of lipoplex delivery resulted in marked changes to detectable specific immune responses suggesting improvements in antibody generation or CD4+ T cell priming. Each of the single dose formulations showed a modest (but not statistically significant) control of bacterial load in challenged mice. Whilst the lack of a robust protective effect is disappointing it is unsurprising as with the exception of a Cu/Zn SOD plasmid delivered directly to the spleen[17], demonstrable protective efficacy against brucellosis has yet to be demonstrated from a single inoculation with a DNA vaccine.

The lipoplex strategy appears to have been successful in boosting the humoral immune responses elicited by both candidate vaccines. In the case of the *omp25 *vaccines this was seen as an increase in specific IgG titre and the number of responsive animals observed in the single dose lipoplexed p-*omp25 *group compared to the single dose naked DNA group. For the p-*ialB *vaccine specific antibodies were not detected from a single dose with naked DNA but lipoplex delivery resulted in measurable specific IgG1 from 80% of the mice. Thus, demonstrating that lipoplex delivery was able to increase the immunogenicity of the candidate vaccines and deliver a stronger or detectable response after a single immunisation. Similar improvements to antibody generation through use of liposomes to deliver DNA vaccines have been reported by Perrie [28] and more recently by Hiszczyńska-Sawicka [29]. The relevance of antibodies for clearance of *Brucella *remains undetermined at this point. Defining the role of the specific antibodies in the protective effect was outside the remit of this study, and more generally although antibodies are a significant component of the immune response to natural infection they are not considered to be protective.

IFNγ is understood to be the key effector in control of brucellosis in both the murine model [30] and target species [31]. Our findings indicate that antigen specific IFNγ was produced by both CD4+ and CD8+ T cells in response to IalB and Omp25 antigens. The protective multi-dose regimens elicited similar total numbers of antigen specific IFNγ effector cells (around 75 ΔSFC per million) for both candidates. For the *ialB *based vaccines both the single dose strategies produced relatively low quantities of IFNγ secreting T cells compared with the protective boosting strategy. Therefore the relationship between boosting and protection appears to be simple with a threshold of priming reached during the multiple administrations that was not achieved by a single dose. Whether this is due to increased input of antigen or temporal development of the response was not determined in this study. Notably, the lipoplex delivery did result in a modest increase in the number of antigen specific IFNγ secreting cells in comparison with the single dose naked DNA. The demonstration of post-challenge IalB specific responses further supports the notion that the vaccine primes cellular responses. Overall the IalB data suggested that a single dose of naked DNA is capable of priming T cell responses and lipoplexing can improve upon the priming effect.

Interestingly, for the p-*omp25 *candidate lipoplex delivery does not appear to result in a direct improvement of T cell priming. The number of antigen specific T cells elicited by p-*omp25 *was not significantly different (p > 0.05) between single and multi-dose naked DNA or between lipoplex and naked DNA single dose administrations, suggesting that neither boosting nor lipoplexing were able to quantitatively improve T cell priming for this candidate.

In addition to looking for quantitative differences in T cell priming capacity between the various vaccines we also sought to characterise the basic phenotypes of cells involved in the immune response. Both CD4+ and CD8+ T cells have been shown to contribute to the control of *Brucella *growth in the BALB/c mouse in adoptive transfer studies [32] and both are implicated in the control of *Brucella *infection in ruminant species [31]. Additional studies involving *in vivo *depletion strategies have indicated that the involvement of CD8+ cells is crucial in mice [33-35], and that passive transfer of IFNγ secreting CD4+ cells from mice immunised with live vaccines can protect naïve mice against challenge [35,36]. Available data therefore suggests that both cell types have a role to play in control of brucellosis, and therefore the basic phenotypic composition of the vaccine induced immune response was measured in this study to determine whether particular cell subsets were responsible for the protective effect. Although the total number of responder cells was similar for both protective vaccination strategies, the balance of CD4+/CD8+ responding cells was different with the p-*ialB *group response dominated by CD4+ T cells and the p-*omp25 *group response dominated by CD8+ T cells.

Interestingly, the lipoplex delivery of p-*omp25 *altered the profile of responsive T cells from a mainly CD8+ T cell response to a CD4+ dominated response, suggesting a possible effect of lipoplex delivery was to favour or augment CD4+ T cell priming. Indeed, increased CD4+ mediated production of IFNγ has previously been reported as a consequence of using liposome-DNA complexes as an adjuvant for genital herpes vaccines [37] and for delivery of a mycobacterial hsp65 DNA vaccine [38]. Unfortunately, the relative merits of improved CD4+ priming (or decreased CD8+ priming) by the lipoplex approach cannot be determined in this study, since neither the CD8+ dominated naked DNA singly dosed animals nor the CD4+ dominated lipoplex singly dosed animals were able to promote protection. Similarly, the relative contribution of CD4+ and CD8+ T cells for the single dose p-*ialB *vaccines could not be measured in these studies, and therefore whether CD4+ or CD8+ responses are more important for the development of a protective response to this candidate cannot be deduced from this study. Overall, our assessment of T cell responses suggests that the administration of lipoplexed DNA appears to favour CD4+ priming and antibody generation, and therefore additional strategies to improve CD8+ priming may be required.

A direct comparison of our two candidates suggests that Omp25 is a more immunogenic antigen than IalB. Omp25 is an immunogenic protein which is recognised by the sera of infected and convalescent animals. Our own studies [12] suggested that approximately 96% of *B. melitensis *infected goats produce specific antibody against this protein. It shares considerable homology with the Omp31 antigen and our results indicate that our p-omp25 vaccine elicits a similar response to that observed to the pCI-omp31 plasmid vaccine [9]. Notably the role of IalB in *Brucella *virulence and pathogenicity remains undefined. This antigen was shown to be expressed [39] by both virulent *B. melitensis *16 M and the vaccine strain Rev.1, and it bears significant homology to the IalB gene of *B. bacilliformis *which is involved in the process of invasion of erythrocytes for this pathogen [40]. The protein is immunogenic: specific antibody against this protein is detectable in sera from infected sheep and goats (~76%) [12], but its role in Brucella pathogenesis remains undefined at this stage.

Both forms of single dose p-*omp25 *give rise to a high number of IFNγ secreting cells and both IgG1 and IG2a antibodies, whereas p-*ialB *does not give as notable a response after a single inoculation unless lipoplexed. Antigen specific differences such as the presence or absence of secretory signals will influence the *in vivo *expression and consequent presentation to the immune system. For example the presence of a secretory signal in the *ialB *gene is likely to result in better presentation to CD4+ T cells, although this has not been fully investigated in these studies. Furthermore the plasmid structure may influence antigen presentation. Notably, the *ialB *based vaccine is based solely on one type of plasmid backbone, whereas the *omp25 *vaccine is a mixture of two plasmid constructs. Studies with the pTargeT backbone were initially undertaken because this plasmid is designed to have improved expression capacity. However, previous *in vitro *expression studies with the two separate constructs did not reveal any difference in the ability of either construct to express the Omp25 protein. Moreover, in the current study the protective efficacy and total T cell response of mice receiving either candidate vaccine in a multi-dose strategy were similar despite the presence of the pTargeT backbone in one candidate vaccine and not the other. However, the differences in the T cell subsets contributing to this protective response (p-*ialB *vaccinated animals elicited mainly CD4+ antigen specific T cells and p-*omp25 *vaccinated animals produced mainly CD8+ antigen specific T cells) may be related to the presence or absence of the pTargeT backbone in the formulation. Others have shown that the plasmid backbone, through presence of different CpG motifs and/or Intron elements can have a significant effect on the outcome of DNA vaccination (for review see [41]). Further study to determine the influence of the plasmid backbone and the nature of antigen presentation and T cell priming from both the IalB and Omp25 candidate vaccines may be useful in guiding further development of these vaccines and identifying appropriate delivery or adjuvanting strategies.

In the recent study by Rosada *et al *[38] a similar approach to DNA vaccine delivery was assessed whereby cationic liposomes were complexed with the DNA-Hsp65 candidate and demonstrated to engender protection against *M. tuberculosis *in a mouse model. Similarly to our findings, the liposomised DNA was effective in promoting specific immune responses, but a single dose of the vaccine was not protective when delivered intramuscularly. However, significant protective efficacy against intratracheal challenge was observed when it was delivered intranasally suggesting a potent role for a more localised protective effect. This raises the possibility that alternate delivery routes for our lipoplex vaccines may prove beneficial for protecting against more naturally acquired forms of brucellosis (eg: intranasal, oral and aerosol forms of challenge), but this remains to be demonstrated experimentally. Alternate liposome formulations may also be beneficial. Singha *et al *[42] have demonstrated successful improvement of immunogenicity and protective efficacy for a Cu/Zn SOD based DNA vaccine through encapsulation in an *E. coli *lipid based liposome termed an Escherichiosome.

## Conclusions

In conclusion, our study has demonstrated an improvement in humoral immunity through delivery of the plasmids surface adsorbed to cationic liposomes. Lipoplexing resulted in increased antibody titres for both *ialB *and *omp25 *vaccines compared to the equivalent single dose naked DNA vaccine. The effect on the cellular immune response was more subtle, with lipoplex p-*ialB *having a noticeable immunopotentiating effect, but lipoplexed p-*omp25 *contributing to a change in the dominant phenotype of responsive cells. Overall, the data suggest that the liposome formulation is beneficial for promotion of CD4+ T cell responses and antibody generation. The properties of this liposome formulation may benefit vaccine development projects where antibodies and CD4+ T cells are the principal mediators of the protective effect. In terms of *Brucella *vaccine development aims, whilst the liposome delivery effect is significant it is unfortunately insufficient to protect against challenge with virulent *Brucella *and further work is necessary to develop these vaccines to the point where single dose delivery strategies are effective. In particular, since CD8+ T cells are essential in *Brucella *control an assessment of cytotoxic effectors, and methods to augment CD8+ priming would be advocated for future investigations. Simple experiments to determine the effectiveness of the naked DNA canidates delivered in fewer than four doses, delivery in a prime-boost formulation with homologous recombinant protein, or delivery of the two vaccines in a combination formulation, may also assist with determining the potency of these vaccines and to demonstrate areas for potential improvement. Further investigation and possible refinement of the nature of adsorption of the DNA to the liposomes are also warranted. These studies were outside the remit of the described investigation, but are certainly points for consideration in the future. Overall, further analysis of the development, kinetics, longevity and sustainability of the response to Omp25 and IalB is required before real progress can be made toward protective efficacy from a single dose of these promising vaccine candidates.

## Competing interests

The authors declare that they have no competing interests.

## Authors' contributions

NC: Designed the studies, performed the experiments, analysed and interpreted the data and prepared the manuscript. JB: developed and prepared the liposomes for use in this project, BW: assisted with experimental design and preparation of the manuscript. SS, JS and AM developed the original concepts for this project, assisted with troubleshooting, data analysis and preparation of the manuscript. All authors read and approved the final manuscript.
